# Transthyretin Amyloid Cardiomyopathy: The Role and Reach of SGLT2 Inhibitors

**DOI:** 10.1016/j.jacadv.2025.101726

**Published:** 2025-04-25

**Authors:** Paschalis Karakasis

**Affiliations:** Aristotle University of Thessaloniki, General Hospital Hippokration, Thessaloniki, Greece

Jaiswal et al^1^ provide an intriguing analysis of sodium-glucose cotransporter 2 inhibitors (SGLT2i) in transthyretin amyloid cardiomyopathy (ATTR-CM), suggesting significant reductions in all-cause mortality, major adverse cardiovascular events, and ischemic stroke. However, several limitations within the study warrant closer scrutiny to temper enthusiasm for the findings.

First, while the authors utilized propensity score matching to minimize confounding, their reliance on the TriNetX database inherently limits the granularity of clinical data. Key variables—such as the stage of amyloid cardiomyopathy, baseline cardiac biomarkers, and tafamidis adherence—are absent, making it challenging to assess the true baseline comparability between groups. Without accounting for disease severity, the risk of indication bias persists, as patients prescribed SGLT2i may have inherently different risk profiles or concurrent conditions influencing outcomes.

Second, the comparison between patients receiving SGLT2i and the control group raises questions about the adequacy of matching. While efforts were made to balance baseline characteristics, differences in concurrent treatments, such as tafamidis usage, were not fully addressed. Notably, even after matching, an imbalance in tafamidis use persists, with 40 more treated patients in the SGLT2i group compared to controls. Although the authors report a nonsignificant *P* value of 0.06 for this between-group difference, the utility of this approach has been criticized.^2^

Moreover, the lack of effect on heart failure, atrial fibrillation, and ventricular tachycardia requires critical interpretation. ATTR-CM’s pathophysiology involves complex myocardial infiltration and high filling pressures,^3^ which may not be adequately addressed by SGLT2i alone. This raises the question: are the observed benefits of SGLT2i truly disease-modifying for ATTR-CM, or are they merely reflective of their broader cardiorenal benefits?^4^ The modest reductions in major adverse cardiovascular events and ischemic stroke could stem from systemic vascular effects rather than direct benefits to amyloid cardiopathy.

Finally, while the authors emphasize the study’s large cohort and long-term follow-up as strengths, the generalizability of these findings to other forms of cardiac amyloidosis, including light-chain amyloidosis, remains unexplored. This omission limits the broader applicability of the results to diverse amyloid populations.

In summary, while this study suggests promising associations, it leaves critical questions unanswered. Moving forward, prospective trials must address these gaps, rigorously accounting for disease-specific nuances and potential confounders. Until then, the role of SGLT2i in ATTR-CM remains intriguing but unproven.
